# Low-cost simulator for teaching medication administration to infants: a methodological study

**DOI:** 10.1590/0034-7167-2025-0442

**Published:** 2026-10-16

**Authors:** Marilia Souto de Araújo, Raphael Raniere de Oliveira Costa, Alessandra Mazzo, Fillipi André dos Santos Silva, Soraya Maria de Medeiros

**Affiliations:** I Governo do Estado do Rio Grande do Norte, Secretaria de Saúde. Natal, Rio Grande do Norte, Brazil; II Universidade Federal do Rio Grande do Norte. Natal, Rio Grande do Norte, Brazil; III Universidade de São Paulo. Bauru, São Paulo, Brazil

**Keywords:** Low Cost Technology, Simulation Training, Drug Administration Routes, Validation Study, Nursing., Tecnología de Bajo Costo, Entrenamiento Simulado, Vías de Administración de Medicamentos, Estudio de Validación, Enfermería.

## Abstract

**Objectives:**

to develop and validate a low-cost, low-tech simulator for teaching medication administration to infants.

**Methods:**

a methodological study, with a quantitative approach, conducted in September 2023. In the first phase, a low-cost simulator was planned. In the second, it was developed using accessible materials. Finally, its appearance and functionality were validated by nine judges. Data were analyzed using the Kappa coefficient, the Content Validity Index (CVI), and the Factorial Validity Index (FVI).

**Results:**

a high degree of agreement was achieved among experts, with a Kappa coefficient of 0.99. The CVI was equal to 1 for 96% of the items analyzed, and the FVI indicated a mean acceptance rate of 99%.

**Conclusions:**

the developed simulator proved to be an innovative, reproducible, and low-cost educational technology, representing a more accessible alternative compared to models available on the market.

## RESUMEN

**Objetivos:**

desarrollar y validar un simulador de bajo costo y baja tecnología para enseñar la administración de medicamentos a bebés.

**Métodos:**

estudio metodológico con enfoque cuantitativo, realizado en septiembre de 2023. En la primera fase, se planificó el simulador de bajo costo. En la segunda, se desarrolló con materiales accesibles. Finalmente, su apariencia y funcionalidad fueron validadas por nueve jueces. Los datos se analizaron mediante el coeficiente Kappa, el Índice de Validez de Contenido (IVC) y el Índice de Validez Factorial (IVF).

**Resultados:**

se alcanzó un alto grado de acuerdo entre los expertos, con un coeficiente Kappa de 0,99. El IVC fue igual a 1 para el 96% de los artículos analizados, y el IVF indicó una tasa de aceptación promedio del 99%.

**Conclusiones:**

el simulador desarrollado demostró ser una tecnología educativa innovadora, reproducible y de bajo costo, representando una alternativa más accesible en comparación con los modelos disponibles en el mercado.

## INTRODUCTION

Low-cost simulators are technologies geared towards clinical simulation, developed from accessible, alternative, and sustainable materials. They are characterized by their simplified assembly, reproducibility, and significantly lower cost compared to commercial models, and may present different levels of technological complexity^(1)^. Initially, it is believed that such devices emerged in developing countries as a viable alternative to fill gaps in the teaching-learning process, without compromising the quality of education^(2)^.

In Latin America, there is a growing trend towards the development and use of low-cost simulators^(3)^. Among the main factors driving this practice are the challenges related to the financing of educational technologies, the high cost of acquiring commercial simulators, and the need for specific models for training certain skills. These barriers often result in limited access to such resources, leading to educational inequalities between institutions and territories^(4)^.

Several advantages are attributed to the use of low-cost simulators, including reproducibility, stimulation of creativity, economic viability, the possibility of developing specific clinical skills, and the expansion of learning opportunities^(5)^.

The literature also indicates that the use of these devices is associated with improved student performance, greater content retention, reduced patient exposure to risks, and easier teaching resource maintenance^(6,7)^.

In the field of health education, simulators play a strategic role for both students and teachers, contributing to developing technical, motor, and cognitive skills, as well as clinical reasoning and manual dexterity.

These practices are carried out in controlled, safe, and risk-free environments for patients, with the aim of improving care and strengthening patient safety^(8)^. When integrated into the learning process, these experiences promote greater motivation, engagement, and autonomy among students, fostering self-learning and strengthening self-efficacy^(9)^.

Among the most recurrent adverse events in nursing practice, medication errors stand out as a problem of global relevance^(10,11)^. According to the Food and Drug Administration, these events result, on average, in one death per day in the United States^(12)^. Given this reality, national and international organizations have advocated for professional qualification as a priority strategy for preventing these errors and promoting a safer and more effective care^(13,14)^.

In this scenario, it is urgent to develop educational technologies that strengthen the teaching and training of students and healthcare professionals^(15)^. In particular, the importance of practical training in medication administration to infants stands out, considering the vulnerability of this age group and the increasing incidence of incidents and harm related to medication practice.

## OBJECTIVES

To develop and validate a low-cost, low-tech simulator for teaching medication administration to infants.

## METHODS

### Ethical aspects

The study was conducted in accordance with national and international ethical guidelines. It was approved by the *Universidade Federal do Rio Grande do Norte* Research Ethics Committee, whose opinion is attached to this submission. All participants signed the Informed Consent Form.

### Study design, period, and place

This is a methodological study focused on educational technology construction and validity. The process involved three main stages: planning (feasibility analysis, project design and structuring); development (materials survey, price research and prototype assembly); and finally, technology testing and validity.

Data collection took place in September 2023 at a federal public university in the state of Rio Grande do Norte.

This manuscript was written following Guidelines for Reporting Reliability and Agreement Studies (GRRAS) recommendations, which guides the transparent description of reliability and validity studies^(16)^. Thus, methodological design, participant/judge selection, data collection and analysis procedures, as well as statistical coefficients employed (Kappa, Content Validity Index (CVI), and Factorial Validity Index (FVI)) were detailed, in accordance with the items foreseen in the GRRAS checklist^(16)^.

### Population, inclusion and exclusion criteria

Judges were selected by convenience from among faculty and staff of the federal public university of Rio Grande do Norte linked to the technical nursing course or undergraduate nursing program. The inclusion criteria adopted for participants were a minimum score based on previously defined and structured criteria as follows: doctoral degree in the field of study (three points); master’s degree in the field of study (two points); specialist degree in the field of study (one point); teaching experience in child health (two points); teaching experience in semiology and semiotics (two points); use of simulators in their practices (three points); and publications related to simulation (two points). Those who were absent from work due to medical leave, maternity/paternity leave, vacation, or other formal absences during the data collection period were excluded.

In total, nine evaluators participated, a number higher than the minimum of six recommended by Pasquali^(17)^. For selection, the Fehring validity model^(18)^ was adopted, with adjustments, considering criteria such as academic qualifications, area of expertise and experience in simulation.

### Study protocol

In the first stage of this study, during low-cost simulator planning, the initial concept was to develop a simulator based on a full-body mannequin with movable joints, in black and the size corresponding to an infant, intended for training in intravenous, intramuscular, and subcutaneous medication administration. To support the creative process, an initial prototype design was created using Microsoft Paint software, available on the Microsoft Windows^®^ operating system.

To enable intravenous route training, the initial prototype design was inspired by two studies that guided the creation of anatomically positioned veins, made with latex tubes (tourniquets) to simulate the texture and resistance of veins^(19,20)^. For subcutaneous and intramuscular administration, silicone-coated foams were used, according to the methodology described by Silva *et al*.^(21)^.

The next stage in developing a low-cost simulator involved researching materials and costs through reading articles and contacting specialized companies via email and telephone. A market survey was also conducted to identify the prices of simulators that met the following requirements: 1) enabling subcutaneous, intravenous, and intramuscular medication administration; 2) enabling skin antisepsis; 3) allowing liquid injection; 4) enabling the creation of skin folds. It is important to note that this market research served to identify existing models and compare their costs. Based on this, a prototype simulator was built using low-cost materials that ensured compliance with the above-mentioned recommendations.

The search for materials took place both in person, in the researcher’s city, and online, on sales websites. Based on references, videos, and other sources, various materials were selected for testing. The setup began with an analysis of the mannequin, on which precise markings were made for the incisions. For the intravenous area, the markings were made in the cubital fossae of both arms, considering the small size of the mannequin’s extremities, which made larger incisions unfeasible.

In the case of the subcutaneous area, the umbilical scar, absent in the original mannequin, was marked, thus delimiting the area to be cut. For intramuscular administration, the vastus lateralis muscle of the thigh was chosen because it is thick, well-developed, and has a lower risk of hitting nerves or large blood vessels, as recommended for intramuscular application in infants^(22)^.

After the incisions, n^o^ 200 latex tubes (tourniquets) were inserted to simulate veins, and foam was used to fill the thighs, arms, and abdomen. The idea of using foams with different densities was to increase fluid absorption and extend the simulator’s lifespan. The foams were fixed with glue, ensuring stability even during the skin folding process.

To simulate skin, liquid silicone rubber was used, mixing 500 g of silicone and 20 g of catalyst. An attempt was made to color the material using aniline powder; although the desired color was achieved, it did not reach the desired black tone, resulting in gray due to the white base of the silicone. After 24 hours of curing, the pieces were demolded, cut, and tested for bonding to the mannequin. Several adhesives were assessed until acetic silicone showed the best result, even with technical indications restricting its use to specific plastics. To ensure fixation, the simulator was wrapped in plastic film during the 24-hour drying period. Finally, a child’s garment was acquired to give the simulator a more realistic appearance.

Validity of the appearance of low-cost simulators concerns the assessment of the aesthetic composition (colors, shapes, and proportions) and its coherence with the content it proposes to represent^(23)^. Although appearance validity is frequent, there is a lack of specific theoretical frameworks for this type of study in nursing, often being related to content validity^(23,24)^. Therefore, the authors developed their own instrument for assessment, considering the lack of validated instruments aimed at low-cost simulators.

Finally, to validate functionality, defined by the Aurélio Brazilian dictionary^(25)^ as the ability to adequately perform the function for which it was created, judges assessed whether the simulator met the objective of enabling subcutaneous, intramuscular, and intravenous medication administration.

During the tests, judges administered the medications and assessed whether the simulator fulfilled its purpose, using a psychometric scale developed by the authors, based on the Likert scale from 1 to 5, with scores of 4 and 5 being considered positive^(26)^.

### Analysis of results and statistics

The data collected from the printed forms were compiled into a database using Microsoft Excel 365^®^ in a double-entry process by two different researchers, in order to guarantee the reliability of the record. Subsequently, the database was transferred for processing in the Statistical Package for the Social Sciences version 23 software (IBM Corporation, Armonk, NY, USA).

The analysis of results included the calculation of: Kappa coefficient, used to measure the degree of agreement among judges; CVI, to measure the proportion of judges who considered each item adequate for the construct; and FVI, which verifies whether the items are adequately loaded onto the instrument’s theoretical factors (construct validity), thus ensuring a statistical assessment of the validity process.

### Access to original data and research relevance

This manuscript forms part of a doctoral thesis entitled “*Construção, validação e avaliação da eficácia de um simulador de baixo custo voltado para o ensino de administração de medicamentos em lactentes*”^(16)^, available in full in the *Universidade Federal do Rio Grande do Norte*’s institutional repository: https://repositorio.ufrn.br/handle/123456789/57627. Access to the document allows consulting the original data, detailed records of the construction stages, and preliminary versions of the instruments used.

This research is relevant to nursing by proposing an innovative, low-cost, reproducible educational technology adapted to the needs of teaching medication administration to infants. By detailing the simulator’s construction and validity process, the study contributes to expanding access to simulation practices in institutions with limited resources, promoting the training of better-prepared professionals aligned with national and international patient safety recommendations.

## RESULTS

Table 1 describes the materials used to construct the simulator, their individual and total cost, the place of acquisition, the type of material, and the exchange rate for the period. All items were purchased from local businesses in the city of Natal, Rio Grande do Norte, between March and April 2023, a period in which the average commercial dollar exchange rate was US$1.00 = R$5.06.

**Table 1:** Materials used for constructing a simulator, Natal, Rio Grande do Norte, Brazil, 2023

Items	Unit	Value (in reais)	Value in US dollars^*^
Full-body mannequin, lactating size	1	R$ 150.00	US$ 29.61
Silicone with Sigmol® catalyst	1	R$ 50.00	US$ 9.87
One meter of laminated foam, density 20, 2 cm thick	1	R$ 30.00	US$ 5.92
One meter of laminated foam, density 33, 2 cm thick	1	R$ 24.00	US$ 4.74
High-precision digital scale	1	R$ 25.00	US$ 4.93
One tube of transparent acetic silicone 280 g	1	R$ 10.00	US$ 1.97
One roll of plastic wrap	1	R$ 8.00	US$ 1.58
Black aniline powder dye	1	R$ 7.00	US$ 1.38
Red aniline powder dye	1	R$ 7.00	US$ 1.38
Brascoplast contact adhesive	1	R$ 7.00	US$ 1.38
Utility knife	1	R$ 5.00	US$ 0.99
Tourniquets		R$ 4.50	US$ 0.89
Intravenous fluid administration set	1	R$ 3.00	US$ 0.59
**Total value**		**R$ 326.20**	**US$ 326.20**

* *US dollar exchange rate in April 2023: US$5.06.*

The mannequin used was a full-body, infant-sized mannequin made of rigid plastic with a flexible PVC outer coating, which allowed for the adaptation of structures simulating tissues and venous access. For artificial skin covering and production, silicone with catalyst (Sigmol^®^ base) and laminated foam of densities 20 and 33 were used; these materials are easy to handle and low-cost.

The cost of the materials needed for the structural construction of a simulator alone was R$326.20 (US$54.39). However, considering the additional expenses for supplies used during testing, improvements, and adjustments until the final functional version, the total cost was R$963.00 (approximately US$190.32). Therefore, the actual cost of developing the prototype, including construction and testing, was significantly higher than the cost of the initial materials, which is relevant for professionals interested in reproducing the simulator in other scenarios.

Figure 1a shows the different stages of the simulator’s development: from the initial design (graphic representation) to the characterization process with children’s clothing. It is important to note that no gender characteristics or facial expressions were included in the simulator.

**Figure 1:**
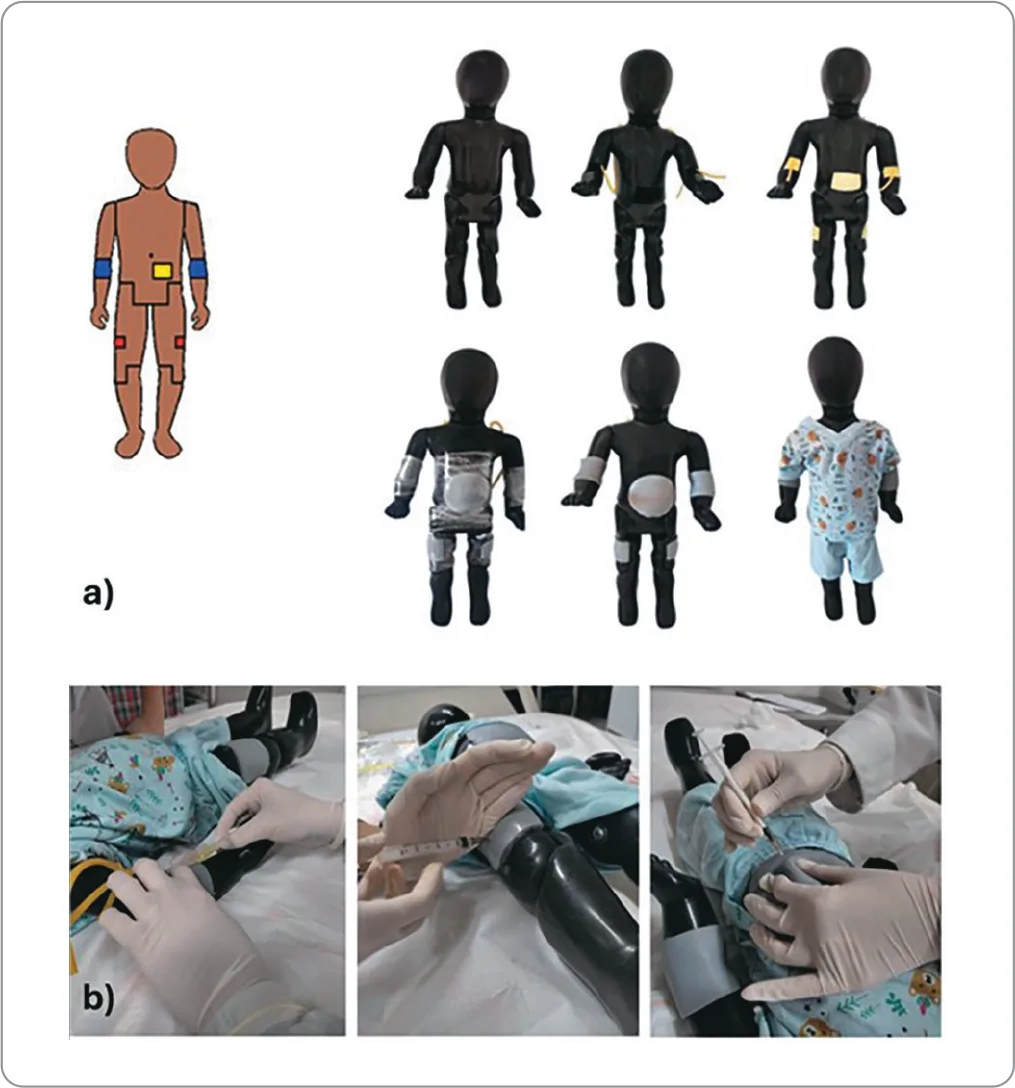
Construction of a low-cost simulator and demonstration of intravenous, intramuscular, and subcutaneous drug administration, Natal, Rio Grande do Norte, Brazil, 2023

### Simulator appearance and functionality validity

The validity phase involved nine judges, experts in the study’s subject matter, and was conducted at the Nursing Department of the institution under study. A private room was set up to provide a detailed explanation of the entire simulator construction process. Following the presentation, the prototype remained available for assessment regarding its functionality, appearance, and other general aspects.

The majority of participants were women (77.78%), with a doctoral degree (66.67%) and working as professors (88.89%). Also noteworthy is the judges’ prior experience in pediatric nursing (77.78%), medication administration (77.78%), and clinical simulation (100%).

Analysis of the 27 assessed items indicated a high level of agreement among judges, with a Kappa coefficient of 0.99, reflecting excellent agreement (Table 2). In relation to the CVI, 96% of items (n = 26) obtained a CVI equal to 1, indicating full agreement. Only the item referring to “Intravenous drug administration” showed an agreement index of 89%.

**Table 2:** Results of the Kappa coefficient, Content Validity Index, and Factorial Validity Index obtained by the evaluators during the validity process of the simulator’s functionality, Natal, Rio Grande do Norte, Brazil, 2023

Items	n	Kappa	CVI	FVI
1. Intravenous drug administration	8	0.78	0.89	0.89
2. Subcutaneous drug administration	9	1.00	1.00	1.00
3. Intramuscular drug administration	9	1.00	1.00	1.00
4. Performing skin antisepsis	9	1.00	1.00	1.00
5. It allows positioning needles at a 30° angle	9	1.00	1.00	1.00
6. It allows positioning needles at a 45° angle	9	1.00	1.00	1.00
7. It allows positioning needles at a 90° angle	9	1.00	1.00	1.00
8. Performing a subcutaneous fold	9	1.00	1.00	1.00
9. Releasing the subcutaneous fold	9	1.00	1.00	1.00
10. Inserting needles at a 30° angle	9	1.00	1.00	1.00
11. Inserting needles at a 45° angle	9	1.00	1.00	1.00
12. Inserting needles at a 90° angle	9	1.00	1.00	1.00
13. Introduced with a single, quick, and resistance-free movement at a 30° angle	9	1.00	1.00	1.00
14. Introduced with a single, quick, and resistance-free movement at a 45° angle	9	1.00	1.00	1.00
15. Introduced with a single, quick, and resistance-free movement at a 90° angle	9	1.00	1.00	1.00
16. Administration of fluids intravenously	9	1.00	1.00	1.00
17. Subcutaneous fluid administration	9	1.00	1.00	1.00
18. Intramuscular fluid administration	9	1.00	1.00	1.00
19. Needle should be withdrawn with a single, smooth movement for intravenous administration	9	1.00	1.00	1.00
20. Needle should be withdrawn with a single, smooth movement for subcutaneous administration	9	1.00	1.00	1.00
21. Needle should be withdrawn with a single, smooth movement for intramuscular administration	9	1.00	1.00	1.00
22. Local pressure on the injection site after venipuncture	9	1.00	1.00	1.00
23. Local pressure on the injection site after subcutaneous injection	9	1.00	1.00	1.00
24. Local pressure on the injection site after intramuscular injection	9	1.00	1.00	1.00
25. Intravenous drug administration training	9	1.00	1.00	1.00
26. Subcutaneous drug administration training	9	1.00	1.00	1.00
27. Intramuscular drug administration training	9	1.00	1.00	1.00
**Total**		**0.99**	**0.99**	**0.99**

*n - absolute frequency; Kappa - Kappa coefficient; CVI - Content Validity Index; FVI - Factorial Validity Index.*

The results obtained indicated that all assessed items presented satisfactory indices, with an average FVI of 99% agreement among judges. The exception was the item “Intravenous drug administration”, which obtained an FVI of 0.89. Figure 1b illustrates the developed simulator, highlighting its main functionalities.

Table 3 presents the results of the Kappa coefficient, CVI, and FVI, obtained by the evaluators during the validity process of the simulator’s functionality for overall assessment.

**Table 3:** Agreement among judges using the Kappa coefficient, Content Validity Index, and Factorial Validity Index, Natal, Rio Grande do Norte, Brazil, 2023

Items	n	Kappa	CVI	FVI
1. It can be reused	9	1.00	1.00	0.89
2. It can be easily transported	9	1.00	1.00	1.00
3. It can be used in different learning spaces (classroom, laboratory, home etc.)	9	1.00	1.00	1.00
4. It can be used in virtual learning	8	0.78	0.89	0.89
5. The assessed simulator is easy to manufacture	9	1.00	1.00	1.00
6. It trains skills in both students and healthcare professionals	9	1.00	1.00	1.00
7. Training in a variety of different skills	8	0.78	0.89	0.89
8. It both demonstrates and trains skills	9	1.00	1.00	1.00
9. It can be used for research	9	1.00	1.00	1.00
10. No risks for users	9	1.00	1.00	1.00
11. It can be used intuitively	9	1.00	1.00	1.00
12. It can be used after simple explanations	9	1.00	1.00	1.00
**Total**		**0.96**	**0.98**	**0.98**

*n - absolute frequency; Kappa - Kappa coefficient; CVI - Content Validity Index; FVI - Factorial Validity Index.*

The results demonstrated a high degree of agreement among judges regarding the 12 items assessed, with an overall Kappa coefficient of 0.96, indicating excellent agreement. However, the items “It can be used in virtual learning” and “Training in a variety of different skills” showed a moderate Kappa coefficient (0.78).

Regarding the CVI, it was observed that 83% of items (n = 10) achieved a CVI equal to 1, indicating full agreement among the evaluators. The two items mentioned earlier were the only ones with lower agreement, presenting a CVI of 0.89.

Finally, the FVI also indicated that the items were considered acceptable, with an average agreement rate of 98%. Again, the items “It can be used in virtual learning” and “Training in a variety of different skills” obtained an FVI of 0.89.

Table 4 presents the detailed results of the Kappa, CVI, and FVI coefficients, assigned by the judges during the validity process of the simulator’s overall functionality.

**Table 4:** Agreement among judges using the Kappa coefficient, Content Validity Index, and Factorial Validity Index, Natal, Rio Grande do Norte, Brazil, 2023

Items	n	Kappa	CVI	FVI
1. The appearance of the assessed simulator is suitable for students	9	1.00	1.00	1.00
2. The appearance of the assessed simulator is harmonious	9	1.00	1.00	1.00
3. The appearance of the constructed simulator is similar to that of a commercial simulator	8	0.78	0.89	0.89
4. The assessed simulator resembles the appearance of a black child	9	1.00	1.00	1.00
5. The appearance of the assessed simulator is compatible with that of a child	9	1.00	1.00	1.00
6. The assessed simulator has a realistic appearance	9	1.00	1.00	1.00
**Total**		**0.96**	**0.98**	**0.98**

*n - absolute frequency; Kappa - Kappa coefficient; CVI - Content Validity Index; FVI - Factorial Validity Index.*

The analysis of the 12 items assessed by judges revealed a high degree of agreement, with an overall Kappa coefficient of 0.98, indicating excellence in the assessment of most items. The only exception was the item “The appearance of the constructed simulator is similar to that of a commercial simulator”, which showed moderate agreement, with a Kappa of 0.78.

Concerning CVI, 83% of items (n = 10) achieved a CVI equal to 1, demonstrating full agreement among evaluators. The item mentioned earlier obtained a CVI of 0.89, indicating a slight divergence in judges’ perception.

Similarly, the FVI indicated that the items were considered acceptable, with an average agreement of 98%. The item “The appearance of the constructed simulator is similar to that of a commercial simulator” again presented the lowest value, with an FVI of 0.89.

## DISCUSSION

Creating a low-cost simulator involves multiple stages, beginning with identifying a specific educational need, with creativity as a central element permeating the entire development process^(27,28)^.

At the institution where the study was conducted, there were no pediatric simulators that would allow training in medication administration via the three routes described in the research. Therefore, the project arose from recognizing this training gap. In addition, a situational assessment identified that students had specific difficulties administering medication to infants.

A full-body simulator was chosen to encompass a greater number of skills in a single piece of equipment. Although the validity of this study did not directly focus on other practices, the researchers believe it is feasible to use the same simulator for other nursing purposes, such as measuring circumferences (head, chest, and abdomen), measuring weight and length, applying wound molds for dressing training, and even simulating bed baths.

The inclusion of movable joints was designed to give the simulator greater mobility, allowing for different positions during medication administration practice. Moreover, it aimed to enable the use of real liquids, making the training more realistic.

As for color, a black simulator was chosen, considering that, in laboratory environments, students generally only have contact with white models, which does not represent Brazilian children’s ethnic-racial diversity. This gap is pointed out in the study by Bastos, Spadácio, and Matsue^(29)^, which questions to whom these models resemble, since “they are all white, young and thin, without exception”.

Regarding facial features, details such as eyes, mouth, nose, and ears were not included. However, recognizing the importance of representing different physical characteristics and even simulating syndromic faces, the authors suggest the use of self-adhesive stickers to meet this demand.

Concerning functionality, the simulator achieved excellent agreement rates among judges, presenting an overall Kappa coefficient of 0.96, a CVI of 0.98, and an FVI of 0.98. These same values were also found for criteria related to appearance.

In the item “Intravenous drug administration”, a moderate Kappa coefficient (0.78) was observed, as the evaluators highlighted that the mannequin’s hand position made the procedure difficult. This limitation stems from the structure of the mannequin used. On the other hand, it is understood that this difficulty can be useful in simulating real situations, since inadequate positioning of patients or professionals is recognized in the literature as a factor that compromises the success of peripheral venous puncture^(30,31)^.

To assess costs, research was conducted on six specialized websites, which revealed that there are no simulators capable of simultaneously training subcutaneous, intramuscular, and intravenous routes. The available models generally offer isolated parts, such as arms, skin kits, hands, or buttocks, with prices ranging from US$2.00 to US$625.00.

Therefore, the developed simulator proved to be low-cost, presenting a lower value than commercial models with similar purposes, requiring little maintenance, allowing reuse without frequent replacement of parts, and dispensing with specialized labor for assembly or maintenance.

The materials used in making the simulator are easily accessible, and many can even be obtained through donations, such as mannequins for displaying children’s clothing. The low cost also favors large-scale production, allowing its use in various environments, such as healthcare services and even student residences.

### Study limitations

The number of judges participating in the validity stands out among the limitations. Although it met the minimum number suggested by the literature, the institution had a reduced number of faculty members who met the established criteria for participation in the study.

### Contributions to nursing, health and/or public policy

This study contributes to promoting collaboration competence, as foreseen in the pedagogical model of the United Nations Educational, Scientific and Cultural Organization for education focused on sustainability. This competence involves the ability to cooperate and respect others, even in the face of disagreements^(32,33)^. At the end of the patenting process, the simulator will be donated to the nursing laboratory of the institution where it was developed.

## CONCLUSIONS

Based on assessment results, the low-cost simulator is considered validated in terms of appearance and functionality, allowing for training intravenous, subcutaneous, and intramuscular medication administration skills. It is expected that the detailed construction of the developed low-cost simulator will allow for its reproduction in contexts that lack such a resource for nursing education, contributing to the improvement of the training of students and healthcare professionals.

## Data Availability

The research data are available within the article.

## References

[1] Oliveira SN, Canever BP, Silveira NIR, Fernandes SR, Martini JG, Lino MM. (2019). Low cost peripheral venipuncture simulator: from confection to evaluation. Rev Enferm UERJ.

[2] Bulamba F, Sendagire C, Kintu A, Hewitt-Smith A, Musana F, Lilaonitkul M (2019). Feasibility of simulation-based medical education in a low-income country: challenges and solutions from a 3-year Pilot Program in Uganda. Simul Healthc.

[3] Targino AN, Silva AP, Leitão FNC, Zangirolami-Raimundo J, Echeimberg JO, Raimundo RD. (2021). Low cost simulator for cardiopulmonary unobstructed and reunion procedures in infants. J Hum Growth Dev.

[4] Conselho Regional de Enfermagem de São Paulo (COREN-SP) (2017). Uso seguro de medicamentos: guia para preparo, administração e monitoramento.

[5] Knobel R, Costa RRO., Pereira GA, Guedes HTV (2021). Simulação em saúde para ensino e avaliação: conceitos e práticas.

[6] Tramontin DF, Teixeira RKC, Santos DR, Araújo NP, Costa LVP, Calvo FC (2020). If lives gives you tomatoes, training microsurgery. Rev Bras Oftalmol.

[7] Nunes CP, Kulcheski AL, Almeida PA, Filho ES, Stieven E, Graells XS. (2020). Creation of a low-cost endoscopic flavectomy training model. Coluna.

[8] Consulin MF, Truppel TC, Osternack KT. (2022). Development of a low cost simulator for teaching intraosseous puncture in pediatrics. Espac Saude.

[9] Mani S, Nisha M, Varghese J, Johny S, Mathew A. (2020). Cost-effective innovation of locally assembled mannequins for undergraduate skill development in parenteral drug administration. Indian J Pharmacol.

[10] Mulac A, Hagesaether E, Granas AG. (2022). Medication dose calculation errors and other numeracy mishaps in hospitals: analysis of the nature and enablers of incident reports. J Adv Nurs.

[11] Ashour A, Phipps DL, Ashcroft DM. (2022). Predicting dispensing errors in community pharmacies: an application of the Systematic Human Error Reduction and Prediction Approach (SHERPA). PLoS One.

[12] National Coordinating Council for Medication (NCCM) (2014). United.

[13] Carvalho T, Riegel F, Siqueira D. (2017). Medication errors and patient safety: an integrative review. Rev Preven Infec Saúde.

[14] World Health Organization (WHO) (2021). Global patient safety action plan 2021-2030: towards eliminating avoidable harm in health care.

[15] Adnan AI. (2022). Effectiveness of communication skills training in medical students using simulated patients or volunteer outpatients. Cureus.

[16] Kottnet J, Audigé L, Brorson S, Donner A, Gajewki BJ, Hróbjartsson A (2011). Guidelines for Reporting Reliability and Agreement Studies (GRRAS) were proposed. Int J Nurs Stud.

[17] Pasquali L. (2010). Instrumentação psicológica: fundamentos e práticas.

[18] Fehring RJ., Carrol-Johnson RM, Paquete M (1994). Classification of the nursing diagnoses: proceedings of the tenth conference.

[19] Andersen P, O’Brien S, Cox K. (2016). Developing low-cost, high-fidelity resources for blood glucose measurement and cord blood sampling. Clin Simul Nurs.

[20] Hubner GSS. (2015). Desenvolvimento de um manequim simulador de punção venosa para educação na saúde: da ideia ao protótipo.

[21] Silva JP, Pereira GA, Meska MHG, Mazzo A. (2018). Construction and validation of a low-cost simulator for training patients with diabetes mellitus and/or their caregivers in insulin administration. Esc Anna Nery.

[22] Wong WD. (2011). Fundamentos da enfermagem pediátrica.

[23] Souza ACC, Moreira TMM, Borges JWP. (2020). Development of an appearance validity instrument for educational technology in health. Rev Bras Enferm.

[24] Jesus EB, Esteves AVF, Teixeira E, Medeiros HP, Nascimento MHM, Sabioa VM. (2018). Validation of educational technology on phototherapy to guide family members of icteric neonates. Rev Enferm UERJ.

[25] Ferreira ABH. (2010). Dicionário Aurélio da língua portuguesa.

[26] Coluci MZ, Alexandre NMC, Milani D. (2015). Construction of measurement instruments in the area of health. Cienc Saude Colet.

[27] Araújo MS. (2023). Construção, validação e avaliação da eficácia de um simulador de baixo custo voltado para o ensino de administração de medicamentos em lactentes.

[28] Pereira GA, Guedes HTV. (2021). Simulação em saúde para ensino e avaliação: conceitos e práticas.

[29] Bastos RL, Spadacio C, Matsue RY. (2019). In: Reunião de Antropologia da Ciência e da Tecnologia.

[30] Srikanth KK, Lotfollahzadeh S. (2023). In: StatPearls.

[31] Psaila J, Parsons TF, Hahn SA, Fichera L. (2023). Prospective study evaluating whether standard peripheral intravenous catheters can be used for blood collection throughout hospital stay. J Infus Nurs.

[32] Steuernagel CR. (2024). Sustainability: a new paradigm in healthcare education. Cogitare Enferm.

[33] UNESCO (2018). Policy brief.

