# Supplementary material for: Low-cost simulator for teaching medication administration to infants: a methodological study
Source: Rev Bras Enferm. 2026 Oct 16;79:e20250442. doi: 10.1590/0034-7167-2025-0442 (PMC13679173; doi:10.1590/0034-7167-2025-0442)
Supplement: Translation

# Translation: Simulador de baixo custo para ensino de administração de medicamentos em lactentes: estudo metodológico

**DOI:** 10.1590/0034-7167-2025-0442pt

**Authors:** Marilia Souto de Araújo, Raphael Raniere de Oliveira Costa, Alessandra Mazzo, Fillipi André dos Santos Silva, Soraya Maria de Medeiros

**Affiliations:** I Governo do Estado do Rio Grande do Norte, Secretaria de Saúde. Natal, Rio Grande do Norte, Brasil; II Universidade Federal do Rio Grande do Norte. Natal, Rio Grande do Norte, Brasil; III Universidade de São Paulo. Bauru, São Paulo, Brasil

**Keywords:** Tecnologia de Baixo Custo, Treinamento por Simulação, Vias de Administração de Medicamentos, Estudo de Validação, Enfermagem.

## Abstract

**Objetivos:**

desenvolver e validar um simulador de baixo custo e baixa complexidade tecnológica para o ensino da administração de medicamentos em lactentes.

**Métodos:**

estudo metodológico, com abordagem quantitativa, realizado em setembro de 2023. Na primeira fase, o simulador de baixo custo foi planejado. Na segunda, ele foi desenvolvido com materiais acessíveis. Por fim, sua aparência e funcionalidade foram validadas por nove juízes. Os dados foram analisados através do coeficiente Kappa, do Índice de Validade de Conteúdo (IVC) e do Índice de Validade Fatorial (IVF).

**Resultados:**

atingiu-se alto grau de concordância entre os especialistas, com coeficiente Kappa de 0,99. O IVC foi igual a 1 para 96% dos itens analisados, e o IVF indicou uma taxa média de aceitação de 99%.

**Conclusões:**

o simulador desenvolvido mostrou-se uma tecnologia educacional inovadora, reprodutível e de baixo custo, representando uma alternativa mais acessível em comparação aos modelos disponíveis no mercado.

## INTRODUÇÃO

Simuladores de baixo custo são tecnologias voltadas à simulação clínica, desenvolvidas a partir de materiais acessíveis, alternativos e sustentáveis. Caracterizam-se por sua montagem simplificada, possibilidade de reprodução e custo significativamente inferior ao dos modelos comerciais, podendo apresentar diferentes níveis de complexidade tecnológica^(1)^. Inicialmente, acredita-se que tais dispositivos tenham emergido em países em desenvolvimento como alternativa viável para suprir lacunas no processo de ensino-aprendizagem, sem comprometer a qualidade da formação^(2)^.

No contexto latino-americano, observa-se uma crescente adesão ao desenvolvimento e à utilização de simuladores de baixo custo^(3)^. Entre os principais fatores impulsionadores dessa prática, destacam-se os desafios relacionados ao financiamento de tecnologias educacionais, ao alto custo de aquisição de simuladores comerciais e à necessidade de modelos específicos para o treinamento de determinadas habilidades. Essas barreiras resultam, muitas vezes, na limitação do acesso a tais recursos, repercutindo em desigualdades educacionais entre instituições e territórios^(4)^.

Diversas vantagens são atribuídas ao uso de simuladores de baixo custo, incluindo a reprodutibilidade, o estímulo à criatividade, a viabilidade econômica, a possibilidade de desenvolver habilidades clínicas específicas e a ampliação de oportunidades de aprendizagem^(5)^.

A literatura aponta ainda que o uso desses dispositivos está associado à melhoria do desempenho estudantil, à maior retenção de conteúdos, à diminuição da exposição de pacientes a riscos e à facilidade de manutenção dos recursos didáticos^(6,7)^.

No campo da educação em saúde, os simuladores desempenham papel estratégico tanto para estudantes quanto para docentes, ao contribuírem para o desenvolvimento de competências técnicas, motoras e cognitivas, além do raciocínio clínico e da destreza manual.

Essas práticas são realizadas em ambientes controlados, seguros e livres de riscos para o paciente, com o objetivo de qualificar o cuidado e fortalecer a segurança assistencial^(8)^. Quando integradas ao processo formativo, essas experiências promovem maior motivação, engajamento e autonomia entre os discentes, favorecendo a autoaprendizagem e o fortalecimento da autoeficácia^(9)^.

Entre os eventos adversos mais recorrentes na prática de enfermagem, os erros de medicação se destacam como um problema de relevância global^(10,11)^. De acordo com a *Food and Drug Administration*, esses eventos resultam, em média, em um óbito por dia nos Estados Unidos^(12)^. Diante dessa realidade, organismos nacionais e internacionais têm defendido a qualificação profissional como estratégia prioritária para a prevenção desses erros e para a promoção de um cuidado mais seguro e efetivo^(13,14)^.

Nesse cenário, torna-se urgente o desenvolvimento de tecnologias educacionais que fortaleçam o ensino e a capacitação de estudantes e profissionais de saúde^(15)^. Em especial, destaca-se a importância do treinamento prático na administração de medicamentos em lactentes, considerando a vulnerabilidade desse grupo etário e a incidência crescente de incidentes e danos relacionados à prática medicamentosa.

## OBJETIVOS

Desenvolver e validar um simulador de baixo custo e baixa complexidade tecnológica para o ensino da administração de medicamentos em lactente.

## MÉTODOS

### Aspectos éticos

O estudo foi conduzido conforme diretrizes éticas nacionais e internacionais. Foi aprovado pelo Comitê de Ética em Pesquisa da Universidade Federal do Rio Grande do Norte, cujo parecer está anexado à presente submissão. Todos os participantes assinaram o Termo de Consentimento Livre e Esclarecido.

### Desenho, período e local do estudo

Trata-se de estudo metodológico voltado à construção e validação de uma tecnologia educacional. O processo envolveu três etapas principais: planejamento (análise de viabilidade, elaboração do desenho e estruturação do projeto); desenvolvimento (levantamento de materiais, pesquisa de preços e montagem do protótipo); e, por fim, testagem e validação da tecnologia.

A coleta de dados ocorreu no mês de setembro de 2023 em uma universidade pública federal do estado do Rio Grande do Norte.

A redação deste manuscrito seguiu as recomendações do *Guidelines for Reporting Reliability and Agreement Studies* (GRRAS), que orienta a descrição transparente de estudos de confiabilidade e validade^(16)^. Assim, foram detalhados o delineamento metodológico, a seleção dos participantes/juízes, os procedimentos de coleta e análise de dados, bem como os coeficientes estatísticos empregados (Kappa, Índice de Validade de Conteúdo (IVC) e Índice de Validade Fatorial (IVF)), em conformidade com os itens previstos no *checklist* GRRAS^(16)^.

### População, critérios de inclusão e exclusão

Os juízes foram selecionados por conveniência entre docentes e servidores da universidade pública federal do Rio Grande do Norte vinculados ao curso técnico em enfermagem ou graduação em enfermagem. Foram adotados como critérios de inclusão de participantes uma pontuação mínima de seus pontos considerando critérios que foram previamente definidos e estruturados da seguinte forma: título de doutor na área de formação (três pontos); título de mestre na área de formação (dois pontos); título de especialista na área de formação (um ponto); atuação como docente na área da saúde da criança (dois pontos); atuação como docente em semiologia e semiotécnica (dois pontos); utilização de simuladores em suas práticas (três pontos); e publicações relacionadas à temática de simulação (dois pontos). Foram excluídos aqueles que se encontravam em afastamento das atividades por meio de licença médica, licença maternidade/paternidade, férias ou outros afastamentos formais durante o período de coleta de dados.

Ao todo, participaram nove avaliadores, número superior ao mínimo de seis recomendado por Pasquali^(17)^. Para seleção, adotou-se o modelo de validação de Fehring^(18)^, com ajustes, considerando critérios como titulação acadêmica, área de atuação e experiência em simulação.

### Protocolo do estudo

Na primeira etapa do estudo, no planejamento do simulador de baixo custo, incialmente, foi idealizado o desenvolvimento de um simulador baseado em um manequim de corpo inteiro, com articulações móveis, na cor preta e no tamanho correspondente a um lactente, destinado ao treinamento da administração de medicamentos pelas vias endovenosa, intramuscular e subcutânea. Para apoiar o processo criativo, foi elaborado um desenho inicial do protótipo utilizando o *software Microsoft Paint*, disponível no sistema operacional *Windows^®^* da *Microsoft*.

Para possibilitar o treinamento da via endovenosa, a elaboração do desenho inicial do protótipo foi inspirada em dois estudos que orientaram a criação de veias anatomicamente posicionadas, confeccionadas com tubos de látex (garrotes) para simular a textura e resistência das veias^(19,20)^. Quanto às vias subcutânea e intramuscular, foram utilizadas espumas revestidas com silicone, conforme metodologia descrita por Silva *et al*.^(21)^.

A etapa seguinte, no desenvolvimento do simulador de baixo custo, envolveu uma pesquisa de materiais e custos, realizada por meio da leitura de artigos e contato com empresas especializadas, por e-mail e telefone. Também foi feito um levantamento de mercado para identificar os preços de simuladores que atendessem aos seguintes requisitos: 1) promover a administração de medicamentos subcutâneos, endovenosos e intramusculares; 2) viabilizar a realização de antissepsia da pele; 3) possibilitar a injeção de líquidos; 4) propiciar a realização de prega cutânea. Destaca-se que essa pesquisa de mercado serviu para identificar os modelos existentes e comparar os custos. Diante disso, foi construído um protótipo de um simulador com materiais de baixo custo e que garantiu o cumprimento das recomendações acima estabelecidas.

A busca pelos materiais ocorreu presencialmente, na cidade da pesquisadora, e *online*, em sites de venda. A partir de referências, vídeos e outras fontes, selecionaram-se diversos materiais para testes. A montagem começou com a análise do manequim, no qual foram feitas demarcações precisas para os cortes. Para a área endovenosa, as marcações ocorreram nas fossas cubitais de ambos os braços, considerando o tamanho reduzido das extremidades do manequim, que inviabilizava cortes maiores.

No caso da área subcutânea, foi realizada a demarcação da cicatriz umbilical, ausente no manequim original, delimitando-se, assim, a área a ser recortada. Para a via intramuscular, a escolha recaiu sobre a região do vasto lateral da coxa, por ser espessa, bem desenvolvida e com menor risco de atingir nervos ou vasos calibrosos, conforme recomendado para aplicação intramuscular em lactentes^(22)^.

Após os cortes, foram inseridos tubos de látex nº 200 (garrotes) simulando as veias, e espumas foram utilizadas para preencher coxas, braços e abdome. A ideia de utilizar espumas com densidades diferentes buscou aumentar a absorção de líquidos e prolongar a vida útil do simulador. As espumas foram fixadas com cola, garantindo estabilidade mesmo durante a realização da prega cutânea.

Para simular a pele, utilizou-se borracha de silicone líquido, misturando-se 500 g de silicone e 20 g de catalisador. Tentou-se colorir o material usando anilina em pó; embora tenha sido obtida a coloração, não se alcançou o tom preto desejado, resultando em cinza devido à base branca do silicone. Após cura de 24 horas, as peças foram desenformadas, recortadas e testadas para colagem no manequim. Diversos adesivos foram avaliados até que o silicone acético apresentou melhor resultado, mesmo com indicação técnica de restrição para plásticos específicos. Para garantir fixação, o simulador foi envolvido em filme plástico durante as 24 horas de secagem. Por fim, uma roupa infantil foi adquirida para conferir aspecto mais realista ao simulador.

A validação da aparência do simulador de baixo custo diz respeito à avaliação da composição estética (cores, formas e proporções) e sua coerência com o conteúdo que se propõe a representar^(23)^. Apesar de a validação de aparência ser frequente, há carência de referenciais teóricos específicos para esse tipo de estudo em enfermagem, sendo muitas vezes relacionada à validação de conteúdo^(23,24)^. Por isso, os autores elaboraram um instrumento próprio para avaliação, considerando a falta de instrumentos validados voltados a simuladores de baixo custo.

Por fim, para validar a funcionalidade, definida pelo dicionário Aurélio^(25)^ como a capacidade de cumprir adequadamente a função para a qual foi criado, os juízes avaliaram se o simulador atendia ao objetivo de possibilitar a administração de medicamentos pelas vias subcutânea, intramuscular e endovenosa.

Durante os testes, os juízes administraram os medicamentos e avaliaram se o simulador cumpria sua proposta, utilizando uma escala psicométrica desenvolvida pelos autores, baseada na escala Likert de 1 a 5, sendo que as pontuações 4 e 5 foram consideradas positivas^(26)^.

### Análise dos resultados e estatística

Os dados coletados nos formulários impressos foram reunidos em um banco de dados utilizando o *Microsoft Excel 365^®^* em um processo de dupla digitação por dois pesquisadores distintos, com a finalidade de garantir a fidedignidade do registro. Em seguida, o banco de dados foi transferido para tratamento no *software Statistical Package for the Social Sciences,* versão 23 (*IBM Corporation, Armonk, NY, USA*).

A análise dos resultados incluiu o cálculo do: coeficiente Kappa, utilizado para mensurar o grau de concordância entre os juízes; IVC, para medir a proporção de juízes que consideraram cada item adequado para o construto; e IVF, que verifica se os itens estão adequadamente carregados nos fatores teóricos do instrumento (validade do construto), garantindo, assim, uma avaliação estatística do processo de validação.

### Acesso aos dados originais e relevância da pesquisa

Este manuscrito integra a tese de doutorado intitulada “Construção, validação e avaliação da eficácia de um simulador de baixo custo voltado para o ensino de administração de medicamentos em lactentes”^(16)^, disponível integralmente no repositório institucional da Universidade Federal do Rio Grande do Norte: https://repositorio.ufrn.br/handle/123456789/57627. O acesso ao documento permite a consulta aos dados originais, aos registros detalhados das etapas de construção e às versões preliminares dos instrumentos utilizados.

A pesquisa apresenta relevância para a área da enfermagem por propor uma tecnologia educacional inovadora, de baixo custo, reprodutível e adaptada às necessidades do ensino de administração de medicamentos em lactentes. Ao detalhar o processo de construção e validação do simulador, o estudo contribui para ampliar o acesso a práticas de simulação em instituições com recursos limitados, favorecendo a formação de profissionais mais preparados e alinhados às recomendações nacionais e internacionais de segurança do paciente.

## RESULTADOS

Os materiais utilizados para a construção do simulador, seu custo individual e total, o local de aquisição, o tipo de material e a cotação cambial do período estão descritos na Tabela 1. Todos os itens foram adquiridos no comércio local da cidade de Natal, Rio Grande do Norte, entre março e abril de 2023, período em que a cotação média do dólar comercial era de US$ 1,00 = R$ 5,06.

**Tabela 1:** Materiais utilizados para a construção do simulador, Natal, Rio Grande do Norte, Brasil, 2023

Itens	Unidade	Valor (em reais)	Valor em dólares americanos^*^
Manequim de corpo inteiro, tamanho lactante	1	R$ 150,00	US$ 29,61
Silicone com catalisador Sigmol^®^	1	R$ 50,00	US$ 9,87
Um metro de espuma laminada, densidade 20, de 2 cm de espessura	1	R$ 30,00	US$ 5,92
Um metro de espuma laminada, densidade 33, de 2 cm de espessura	1	R$ 24,00	US$ 4,74
Balança digital de alta precisão	1	R$ 25,00	US$ 4,93
Um tubo de silicone acético transparente 280 g	1	R$ 10,00	US$ 1,97
Um rolo de papel filme	1	R$ 8,00	US$ 1,58
Corante em pó de anilina cor preto	1	R$ 7,00	US$ 1,38
Corante em pó de anilina cor vermelha	1	R$ 7,00	US$ 1,38
Cola de contato Brascoplast	1	R$ 7,00	US$ 1,38
Estilete	1	R$ 5,00	US$ 0,99
Garrotes		R$ 4,50	US$ 0,89
Equipo para soro	1	R$ 3,00	US$ 0,59
**Valor total**		**R$ 326,20**	**US$ 326,20**

* *Cotação do dólar americano em abril de 2023: US$ 5,06.*

O manequim utilizado era de corpo inteiro, tamanho lactente, fabricado em plástico rígido com revestimento externo de PVC maleável, o que permitiu a adaptação de estruturas simulando tecidos e acesso venoso. Para o recobrimento e produção da falsa pele, utilizaram-se silicone com catalisador (base Sigmol^®^) e espuma laminada de densidade 20 e 33, materiais de fácil manipulação e baixo custo.

O custo apenas dos materiais necessários para a construção estrutural do simulador foi de R$ 326,20 (US$ 64,39). Entretanto, considerando os gastos adicionais com insumos utilizados durante os testes, aperfeiçoamentos e ajustes até a versão final funcional, o valor total foi de R$ 963,00 (aproximadamente US$ 190,32). Dessa forma, o custo real de desenvolvimento do protótipo, incluindo construção e testagem, foi significativamente maior que o valor dos materiais iniciais, o que é relevante para profissionais interessados em reproduzir o simulador em outros cenários.

A Figura 1a apresenta as diferentes etapas do desenvolvimento do simulador: do desenho inicial (representação gráfica) ao processo de caracterização com vestimenta infantil. É importante destacar que não foram inseridas características de gênero nem expressões faciais no simulador.

**Figura 1:** Construção do simulador de baixo custo e demonstração da administração de medicamentos nas vias endovenosa, intramuscular e subcutânea, Natal, Rio Grande do Norte, Brasil, 2023

### Validação de aparência e funcionalidade do simulador

A etapa de validação contou com a participação de nove juízes, especialistas na temática do estudo, e foi realizada nas instalações do Departamento de Enfermagem da instituição pesquisada. Em uma sala privativa, foi preparado um ambiente apropriado, onde todo o processo de construção do simulador foi apresentado de forma detalhada. Ao término da exposição, o protótipo permaneceu disponível para avaliação quanto à sua funcionalidade, aparência e demais aspectos gerais.

A maioria dos participantes foi composta por mulheres (77,78%), com título de doutorado (66,67%) e atuantes como docentes (88,89%). Destaca-se ainda a experiência prévia desses juízes em enfermagem pediátrica (77,78%), na administração de medicamentos (77,78%) e em simulação clínica (100%).

A análise dos 27 itens avaliados indicou elevado nível de concordância entre os juízes, com um coeficiente Kappa de 0,99, o que reflete excelente concordância (Tabela 2). Em relação ao IVC, 96% dos itens (n = 26) obtiveram IVC igual a 1, indicando concordância plena. Apenas o item referente à “Administração de medicamentos por via endovenosa” apresentou um índice de concordância de 89%.

**Tabela 2:** Resultados do coeficiente Kappa, Índice de Validade de Conteúdo e Índice de Validade Fatorial obtidos pelos avaliadores durante o processo de validação da funcionalidade do simulador, Natal, Rio Grande do Norte, Brasil, 2023

Itens	n	Kappa	IVC	IVF
1. Administração de medicamentos por via endovenosa	8	0,78	0,89	0,89
2. Administração de medicamentos por via subcutânea	9	1,00	1,00	1,00
3. Administração de medicamentos por via intramuscular	9	1,00	1,00	1,00
4. Realização de antissepsia da pele	9	1,00	1,00	1,00
5. Permite posicionar agulhas no ângulo de 30°	9	1,00	1,00	1,00
6. Permite posicionar agulhas no ângulo de 45°	9	1,00	1,00	1,00
7. Permite posicionar agulhas no ângulo de 90°	9	1,00	1,00	1,00
8. Realização de prega subcutânea	9	1,00	1,00	1,00
9. Liberação da prega subcutânea	9	1,00	1,00	1,00
10. Inserção de agulhas no ângulo de 30°	9	1,00	1,00	1,00
11. Inserção de agulhas no ângulo de 45°	9	1,00	1,00	1,00
12. Inserção de agulhas no ângulo de 90°	9	1,00	1,00	1,00
13. Introduzida com um movimento único, rápido e sem resistência no ângulo de 30°	9	1,00	1,00	1,00
14. Introduzida com um movimento único, rápido e sem resistência no ângulo de 45°	9	1,00	1,00	1,00
15. Introduzida com um movimento único, rápido e sem resistência no ângulo de 90°	9	1,00	1,00	1,00
16. Administração de líquidos por via endovenosa	9	1,00	1,00	1,00
17. Administração de líquidos por via subcutânea	9	1,00	1,00	1,00
18. Administração de líquidos por via intramuscular	9	1,00	1,00	1,00
19. Agulha seja extraída com um movimento único e suave na via endovenosa	9	1,00	1,00	1,00
20. Agulha seja extraída com um movimento único e suave na via subcutânea	9	1,00	1,00	1,00
21. Agulha seja extraída com um movimento único e suave na via intramuscular	9	1,00	1,00	1,00
22. Pressão local na área de injeção após o procedimento de punção venosa	9	1,00	1,00	1,00
23. Pressão local na área de injeção após o procedimento de injeção subcutânea	9	1,00	1,00	1,00
24. Pressão local na área de injeção após o procedimento de injeção intramuscular	9	1,00	1,00	1,00
25. Treinamento de administração de medicamentos por via endovenosa	9	1,00	1,00	1,00
26. Treinamento de administração de medicamentos por via subcutânea	9	1,00	1,00	1,00
27. Treinamento de administração de medicamentos por via intramuscular	9	1,00	1,00	1,00
**Total**		**0,99**	**0,99**	**0,99**

*n - frequência absoluta; Kappa - coeficiente Kappa; IVC - Índice de Validade de Conteúdo; IVF - Índice de Validade Fatorial.*

Os resultados obtidos indicaram que todos os itens avaliados apresentaram índices satisfatórios, com um IVF médio de 99% de concordância entre os juízes. A exceção foi o item “Administração de medicamentos por via endovenosa”, que obteve um IVF de 0,89. A Figura 1b ilustra o simulador desenvolvido, destacando suas principais funcionalidades.

A Tabela 3 apresenta os resultados do coeficiente de Kappa, IVC e IVF, obtidos pelos avaliadores durante o processo de validação da funcionalidade do simulador para avaliação geral.

**Tabela 3:** Concordância dos juízes através do coeficiente Kappa, Índice de Validade de Conteúdo e Índice de Validade Fatorial, Natal, Rio Grande do Norte, Brasil, 2023

Itens	n	Kappa	IVC	IVF
1. Pode ser reutilizado	9	1,00	1,00	0,89
2. Pode ser facilmente transportado	9	1,00	1,00	1,00
3. Por ser utilizado em diferentes espaços de aprendizagem (sala de aula, laboratório, domicílio *etc*.)	9	1,00	1,00	1,00
4. Pode ser utilizado no ensino virtual	8	0,78	0,89	0,89
5. Fabricação do simulador avaliado é de fácil aquisição	9	1,00	1,00	1,00
6. Treinamento de habilidades tanto de estudantes quanto de profissionais de saúde	9	1,00	1,00	1,00
7. Treinamento de diversas habilidades diferentes	8	0,78	0,89	0,89
8. Tanto demostra quanto treina habilidades	9	1,00	1,00	1,00
9. Pode ser utilizado para pesquisa	9	1,00	1,00	1,00
10. Sem riscos para quem o utiliza	9	1,00	1,00	1,00
11. Pode ser utilizado intuitivamente	9	1,00	1,00	1,00
12. Pode ser utilizado após simples explicações	9	1,00	1,00	1,00
**Total**		**0,96**	**0,98**	**0,98**

*n - frequência absoluta; Kappa - coeficiente Kappa; IVC - Índice de Validade de Conteúdo; IVF - Índice de Validade Fatorial.*

Os resultados demonstraram um elevado grau de concordância entre os juízes quanto aos 12 itens avaliados, com um coeficiente Kappa geral de 0,96, o que indica excelência na concordância. No entanto, os itens “Pode ser utilizado no ensino virtual” e “Treinamento de diversas habilidades diferentes” apresentaram um coeficiente Kappa moderado (0,78).

Em relação ao IVC, observou-se que 83% dos itens (n = 10) alcançaram um IVC igual a 1, indicando concordância plena entre os avaliadores. Os dois itens mencionados anteriormente foram os únicos com concordância inferior, apresentando um IVC de 0,89.

Por fim, o IVF também apontou que os itens foram considerados aceitáveis, com uma taxa média de concordância de 98%. Novamente, os itens “Pode ser utilizado no ensino virtual” e “Treinamento de diversas habilidades diferentes” obtiveram IVF de 0,89.

A Tabela 4 apresenta os resultados detalhados dos coeficientes Kappa, IVC e IVF, atribuídos pelos juízes durante o processo de validação da funcionalidade geral do simulador.

**Tabela 4:** Concordância dos juízes através coeficiente Kappa, Índice de Validade de Conteúdo e Índice de Validade Fatorial, Natal, Rio Grande do Norte, Brasil, 2023

Itens	n	Kappa	IVC	IVF
1. A aparência do simulador avaliado está adequada para os estudantes	9	1,00	1,00	1,00
2. A aparência do simulador avaliado é harmônica	9	1,00	1,00	1,00
3. A aparência do simulador construído é semelhante à de um simulador comercial	8	0,78	0,89	0,89
4. O simulador avaliado remete à aparência de uma criança preta	9	1,00	1,00	1,00
5. A aparência do simulador avaliado é compatível com uma criança	9	1,00	1,00	1,00
6. O simulador avaliado possui aparência realista	9	1,00	1,00	1,00
**Total**		**0,96**	**0,98**	**0,98**

*n - frequência absoluta; Kappa - coeficiente Kappa; IVC - Índice de Validade de Conteúdo; IVF - Índice de Validade Fatorial.*

A análise dos 12 itens avaliados pelos juízes revelou um alto grau de concordância, com coeficiente Kappa geral de 0,98, o que indica excelência na avaliação da maioria dos itens. A única exceção foi o item “A aparência do simulador construído é semelhante à de um simulador comercial”, que apresentou concordância moderada, com Kappa de 0,78.

Quanto ao IVC, 83% dos itens (n = 10) alcançaram IVC igual a 1, evidenciando concordância plena entre os avaliadores. O item mencionado anteriormente obteve um IVC de 0,89, indicando uma leve divergência na percepção dos juízes.

Da mesma forma, o IVF apontou que os itens foram considerados aceitáveis, com uma média de concordância de 98%. O item “A aparência do simulador construído é semelhante à de um simulador comercial” novamente apresentou o menor valor, com IVF de 0,89.

## DISCUSSÃO

A criação de um simulador de baixo custo envolve múltiplas etapas, que têm início na identificação de uma necessidade educacional concreta, tendo a criatividade como elemento central que permeia todo o processo de desenvolvimento^(27,28)^.

Na instituição onde o estudo foi realizado, não existiam simuladores pediátricos que possibilitassem treinar a administração de medicamentos pelas três vias descritas na pesquisa. Dessa forma, o projeto surgiu ao reconhecer essa carência formativa. Somado a isso, um diagnóstico situacional identificou que os estudantes apresentavam dificuldades específicas ao administrar medicamentos em lactentes.

Optou-se por construir um simulador de corpo inteiro, visando abarcar um maior número de habilidades em um único equipamento. Embora a validação deste estudo não tenha focado diretamente em outras práticas, os pesquisadores acreditam ser viável utilizar o mesmo simulador para outras finalidades de enfermagem, como aferição de circunferências (cefálica, torácica e abdominal), mensuração de peso e comprimento, aplicação de moldes de feridas para treinamento de curativos e até mesmo a simulação de banho no leito.

A inclusão de articulações móveis foi pensada para dar maior mobilidade ao simulador, permitindo diferentes posicionamentos durante a prática de administração de medicamentos. Além disso, havia o objetivo de possibilitar o uso de líquidos reais, tornando o treinamento mais próximo da realidade prática.

Quanto à cor, optou-se por desenvolver um simulador preto, considerando que, nos ambientes de laboratório, os estudantes geralmente têm contato apenas com modelos brancos, o que não representa a diversidade étnico-racial das crianças brasileiras. Essa lacuna é apontada no estudo de Bastos, Spadácio e Matsue^(29)^, que questiona a quem esses modelos se assemelham, visto que “são todos brancos, jovens e magros, sem exceção”.

Sobre as características faciais, optou-se por não inserir detalhes como olhos, boca, nariz e orelhas. Contudo, reconhecendo a importância de representar diferentes características físicas e até mesmo simular faces sindrômicas, os autores sugerem o uso de adesivos autocolantes para atender a essa demanda.

Em relação à funcionalidade, o simulador obteve excelentes índices de concordância entre os juízes, apresentando coeficiente Kappa geral de 0,96, IVC de 0,98 e IVF de 0,98. Esses mesmos valores foram encontrados também para os critérios relacionados à aparência.

No item “Administração de medicamentos por via endovenosa”, observou-se um coeficiente Kappa moderado (0,78), já que os avaliadores destacaram que a posição da mão do manequim dificultava o procedimento. Essa limitação decorre da estrutura do manequim utilizado. Por outro lado, entende-se que essa dificuldade pode ser útil ao simular situações reais, uma vez que o posicionamento inadequado do paciente ou do profissional é reconhecido na literatura como fator que compromete o sucesso da punção venosa periférica^(30,31)^.

Para avaliação do custo, foi realizada pesquisa em seis sites especializados, que revelou não haver simuladores capazes de treinar, simultaneamente, as vias subcutânea, intramuscular e endovenosa. Os modelos disponíveis geralmente trazem partes isoladas, como braços, *kits* de pele, mãos ou glúteos, com preços variando entre US$ 2,00 e US$ 625,00.

Diante disso, o simulador desenvolvido demonstrou ser de baixo custo por apresentar um valor inferior ao dos modelos comerciais de finalidade similar, exigir pouca manutenção, permitir reutilização sem a reposição frequente de peças e dispensar mão de obra especializada para montagem ou conservação.

Os materiais empregados na confecção do simulador são de fácil acesso, e muitos podem ser obtidos até por meio de doações, como manequins para exposição de roupas infantis. O baixo custo também favorece a produção em escala, permitindo o uso em ambientes variados, como serviços de saúde e até mesmo residências de estudantes.

### Limitações do estudo

Entre as limitações, destaca-se o número de juízes participantes na validação. Apesar de ter atendido ao número mínimo sugerido pela literatura, a instituição contava com um número reduzido de docentes que atendiam aos critérios estabelecidos para participação no estudo.

### Contribuições para a área da enfermagem, saúde e/ou políticas públicas

Este estudo contribui para a promoção da competência colaboração, prevista no modelo pedagógico da Organização das Nações Unidas para a Educação, a Ciência e a Cultura para educação voltada à sustentabilidade. Essa competência envolve a capacidade de cooperar e respeitar o outro, mesmo diante de divergências^(32,33)^. Ao final do processo de patente, o simulador será doado ao laboratório de enfermagem da instituição onde foi desenvolvido.

## CONCLUSÕES

Conforme os resultados das avaliações, considera-se que o simulador de baixo custo foi validado quanto à aparência e funcionalidade, permitindo o treino de habilidades de administração de medicamentos pelas vias endovenosa, subcutânea e intramuscular. Espera-se que o detalhamento da construção do simulador de baixo custo desenvolvido permita sua reprodução em contextos que não possuem tal recurso para o ensino de enfermagem, contribuindo para a melhoria da formação de estudantes e profissionais de saúde.

## Data Availability

Os dados de pesquisa estão disponíveis no corpo do artigo.
